# Computational insights on the interplay between electrotaxis and mechanotaxis

**DOI:** 10.1371/journal.pcbi.1014511

**Published:** 2026-07-30

**Authors:** Pablo Sáez, Shardool Kulkarni, Custodio O. Nunes, Min Zhao, Elias H. Barriga

**Affiliations:** 1 Laboratori de Cacùl Numèric (LaCàN), Universitat Politècnica de Catalunya-BarcelonaTech (UPC), Barcelona, Spain; 2 IMTech (Institute of Mathematics), Universitat Politècnica de Catalunya-BarcelonaTech (UPC), Barcelona, Spain; 3 Mechanisms of Morphogenesis Lab, Cluster of Excellence Physics of Life (PoL), TU Dresden, Dresden, Germany; 4 Department of Ophthalmology & Vision Science, School of Medicine, University of California, Davis, Sacramento, California, United States of America; Northeastern University, UNITED STATES OF AMERICA

## Abstract

Understanding how cells migrate in response to external cues has important implications for biology, medicine, and bioengineering. Chemical, mechanical, and electrical signals are the primary drivers of directed cell migration, and each has been extensively studied over the past decades. Among them, chemical cues were the first to be investigated and remain the most widely studied due to their undeniable role in in vivo guidance. Mechanical signals, particularly substrate stiffness gradients, have gained prominence for their ubiquity across cell types and their potential to direct migration. More recently, growing evidence suggests that electrotaxis offers a highly precise and programmable means to guide cell movement. Despite this, these cues are often studied in isolation, whereas in vivo they typically coexist and interact. Using well-established biophysical models, we investigate how mechanical and electrical signals cooperate and how they can be engineered to compete for control over cell migration. Our model shows that an electric field can override and even reverse mechanotaxis. Still, the specific outcomes strongly depend on the cell type or, in other words, on the model parameters that describe how strongly the sensing molecules activate the signaling network. To address this large variability in controlling cell migration, we propose particular steps toward further exploration. To support such future research, we provide a freely available platform for predicting electro-mechanical interactions in cell migration, based on a given cell’s sensing and signaling characteristics, which could tailor the mechanical and electrical signals that arise naturally during organ development, cancer invasion, or tissue regeneration.

## Author summary

Cells move in response to signals in their environment, and this directed movement is essential for processes such as development, tissue repair, and disease progression. We aimed to understand how two important types of signals—mechanical cues from the physical surroundings and electrical cues—work together to guide cell migration. Although both signals have been widely studied separately, much less is known about how they interact when present at the same time, as they are in living systems. We used established theoretical models to examine how mechanical and electrical signals can cooperate or compete in controlling the direction of cell movement. Our model shows that electrical signals can dominate mechanical guidance and, in some parameter regimes, even reverse the direction cells would normally follow. Our results improve understanding of how multiple environmental signals jointly regulate cell behavior. We also provide a freely available predictive platform that may help guide future research on cell migration in tissue regeneration, development, and cancer.

## 1 Introduction

Cell migration is a fundamental biological process that underpins critical aspects of life, including embryonic development, immune responses, wound healing, and pathological conditions such as tumor invasion [1–3]. In both in vivo and in vitro settings, cells respond to various external cues to navigate their environment [4,5]. Moreover, the capacity to guide cellular movement using external stimuli is gaining traction in medicine—aiming to halt tumor invasion, enhance tissue repair, or accelerate wound healing [1,6,7]. In tissue engineering, this control over migration supports the spatial arrangement of cells within engineered constructs, enabling the formation of biomimetic tissues with functional architectures [8,9].

Chemotaxis is among the most well-studied mechanisms governing directed migration, where cells move in response to gradients of soluble biochemical signals [10]. These gradients activate membrane-bound receptors and initiate downstream signaling cascades. Central to this process are the Rho family of GTPases, including RhoA, Rac1, and Cdc42, which regulate myosin and actin cytoskeleton dynamics, as the key drivers of the forces underlying cell migration [11,12]. Specifically, RhoA promotes actomyosin contractility [13–15], Rac1 drives lamellipodia formation [16–19]—aiding in spreading—and Cdc42 controls filopodia formation, essential for environmental sensing and directional probing [20–22]. Cross-talk among these GTPases allows for the spatial coordination of adhesion dynamics, with Rac1 and Cdc42 activity concentrated at the leading edge and RhoA predominantly active at the rear of migrating cells [23,24].

In addition to biochemical cues, mechanical signals are now well recognized as pivotal regulators of cell migration. For instance, cells migrate along gradients of substrate stiffness and adhesion ligand density—phenomena known as durotaxis and haptotaxis, respectively [25–27]. These processes involve asymmetric adhesion forces. Similarly, during frictiotaxis—the migration toward regions of higher extracellular friction—mechanical friction is transduced into intracellular cues that induce polarization of the actomyosin network in integrin-independent migration [28]. Although all these mechanical cues differ in nature, they also share one fundamental aspect: they all induce a front-rear differential friction in the retrograde flow of the cell, which eventually induces a directed cell migration. We will hereafter refer to “mechanotaxis” to encompass durotaxis, haptotaxis, frictiotaxis, and other external cues that create a polarized effective friction transmitted to the retrograde actin flow. Notably, effective friction has been shown to underlie both positive and negative durotaxis, suggesting that friction alone, whether in mesenchymal or amoeboid migration, can drive directed cell movement [29].

While often approached from a mechanical perspective, where cell motility is explained by the balance of forces alone [29–33], durotaxis also engages intracellular signaling pathways similar to those activated in chemotaxis, which directly modify crucial motile forces of the cell for both homeostasis and pathological conditions. Integrin activation, for example, stimulates Rho GTPases, coordinating the organization of adhesion complexes and cytoskeletal remodeling [11,12] and enabling cells to sense and adapt to mechanical forces.

Electrotaxis—the directed migration of cells in response to electric fields (EFs)—is another guidance mechanism observed in physiological contexts such as embryonic development [34] and wound healing [35], and it is present across a wide range of cell types [36–39], and even whole tissues [40,41]. Charged Membrane Proteins (CMPs) such as Epidermal Growth Factor Receptor (EGFR), membrane lipids, and Vascular Endothelial Growth Factor (VEGF) receptors, among others, have been shown to polarize during electrotaxis. Moreover, some of these polarized components may contribute to the asymmetric activation of Rho GTPases, aligning electrotaxis with the same intracellular machinery involved in chemotaxis and durotaxis. Indeed, one of the most fundamental questions in electrotaxis is what specific CMPs are actually responsible for the downstream polarization of GTPases.

In summary, chemotaxis, mechanotaxis, and electrotaxis are all orchestrated through the inside-out and outside-in signaling pathways mediated by Rho GTPases. These directed migration mechanisms occur naturally in vivo and have been extensively studied in isolation. However, in physiological contexts, these cues often coexist, or one can be imposed artificially over the others to foster or arrest their effect in cell migration. Despite this, there is a surprising lack of research exploring how these extracellular signals interact synergistically to regulate cell migration, limiting the potential to efficiently control cell migration.

In this work, we investigate the interplay between mechanical and electrical signals, as easily controllable cues, in directing cell migration. We use well-established computational models to describe and integrate the mechano-chemical phenomena leading to each of these tactic cues. Our aim is to establish a hypothesis for how these cues compete or cooperate to influence the underlying signaling networks and the cell’s motile machinery. To support further research and practical applications, we also present an open-access online platform (available on MATLAB Central File Exchange) that, given the specific characteristics of a cell line, predicts its migratory behavior—including direction, velocity, and intracellular dynamics of Rho GTPases and actomyosin structures.

## 2 Methods

### 2.1 Cell adhesion based on the clutch model

We adopt clutch models to describe how cell adhesion responds to the mechanical properties of the extracellular matrix (ECM). The mathematical formulation, summarized below, follows previous work [42,43]. In this framework, each molecular clutch connects the ECM on one side to the adaptor protein talin on the other. Talin, in turn, links to actin filaments, which are pulled by myosin motors. The actomyosin network generates contractile forces that act through these clutches at adhesion sites.

The velocity of the actin filaments, denoted by $\hat{v}$, follows a force–velocity relationship,


$$\begin{array}{c} \hat{v}=v_{u}\left(1-\frac{F}{F_{stall}}\right), \end{array}$$

where $v_{u}$ is the unloaded actin flow velocity. The stall force of the myosin motors, $F_{stall}$, is defined as $F_{stall}=n_{m}F_{m}$, with $F_{m}$ representing the force required to stall a single myosin motor and $n_{m}$ the number of motors present. The reaction force exerted by the substrate is given by


$$\begin{array}{c} F=\sum_{i=1}^{n_{eng}}F_{c,i}, \end{array}$$

where $n_{eng}$ denotes the number of engaged clutches and $F_{c,i}$ is the force transmitted through the *i*-th clutch.

The force transmitted by each clutch is calculated as


$$\begin{array}{c} F_{c,i}=\kappa_{c}(z_{c,i}-z_{sub}), \end{array}$$

where $\kappa_{c}$ is the stiffness of an individual molecular clutch [43], $z_{c,i}$ is the displacement of the *i*-th clutch, and $z_{sub}$ is the substrate displacement. Once the force on each clutch, $F_{c,i}$, is determined, we compute the corresponding binding and unbinding dynamics. The attachment of the $n_{c}$ clutches to the ECM, representing integrin–ECM interactions, is governed by catch-bond kinetics [44,45]. Clutches bind at a constant rate $k_{on}$ and unbind at a force-dependent dissociation rate $k_{off}$. Mechanosensitivity arises through talin unfolding, which promotes vinculin recruitment and integrin clustering [45]—a process known as adhesion reinforcement. For further details on catch-bond behavior, mechanosensitivity modeling, and parameter values, see [43]. Once the total force *F* is computed, the cell traction is obtained as


$$\begin{array}{c} P=\frac{F}{\pi a^{2}}, \end{array}$$

where $\pi a^{2}$ is the area of a circular adhesion complex.

To compute the dynamics of the clutch model, we perform Monte Carlo (MC) simulations involving repeated stochastic sampling of clutch engagement and disengagement events. These cycles of adhesion formation and rupture are simulated using a constant time step Δt=0.005 s over a total duration of $t_{f}=100$ s, as validated in [43]. The displacement of engaged clutches during each time step is given by $\Delta z=\hat{v}\Delta t$. The substrate displacement at the next time step, $z^{n+1}$, is obtained by solving the force balance between the $n_{eng}$ engaged clutches and the substrate, thereby updating the total force *F*. Simulation outputs are averaged over time to capture the behavior under specific ECM mechanical properties.

Instead of computing the clutch model at each integration point of the Finite Element Model and at each time step (see Section 2.7), we precompute the traction *P* and velocity $\hat{v}$ for each possible value of $n_{c}$ and ECM stiffness. Then, we assume that the effective friction between the cell and the ECM is given by [29,46]


$$\begin{array}{c} \eta =\frac{P}{\hat{v}}, \end{array}$$

This relationship implies that friction varies with ECM stiffness, as with changes in actin velocity and traction force. Under this assumption, strong and stable adhesions—reflected by higher traction forces—correspond to increased friction, whereas weak adhesions produce lower friction, as we discussed in the introduction section.

### 2.2 Electromotility of charged membrane components

The electro-motility of CMPs arises from the interplay between two opposing forces. When a cell is immersed in an electrolyte solution, its negatively charged membrane attracts nearby positive ions, forming a structured region known as the electrical double layer (EDL). Upon the application of an electric field (EF), this EDL is also subjected to the field, generating a fluid movement known as electro-osmotic flow (EOF). CMPs embedded in the cell membrane are dragged by this EOF, enabling their migration along the membrane surface. In addition to being influenced by the global EOF, the charged nature of CMPs causes them to experience local EOF as well as electrostatic interactions. These combined effects constitute electrophoresis, which leads to an asymmetric redistribution of CMPs along the cell membrane [47–49].

To quantify the rate and magnitude of this redistribution—which ultimately drives the downstream polarization of intracellular signaling pathways—we adopted previously established mathematical models [47–50]. In summary, the total electro-motility velocity can be expressed as


$$\begin{array}{c} v_{e}=\frac{\epsilon_{r}\epsilon_{0}E(\zeta_{1}-\zeta_{2})}{\eta_{m}}, \end{array}$$ (1)

where $\epsilon_{r}$ is the relative dielectric constant of the surrounding medium, $\epsilon_{0}$ is the vacuum permittivity, *E* is the strength of the applied EF, and $\eta_{m}$ is the viscosity of the cell membrane. The terms $\zeta_{1}$ and $\zeta_{2}$ represent the ζ-potentials of the CMPs and the cell surface, respectively.

To model the spatiotemporal distribution of positively and negatively charged CMPs, denoted by $\rho_{\pm }$, we use a convection–diffusion equation:


$$\begin{array}{c} \partial_{t}\rho_{\pm }+\partial_{x}\left(v_{e}\rho_{\pm }-D_{M}\partial_{x}\rho_{\pm }\right)=0, \end{array}$$ (2)

where $D_{M}$ denotes the diffusion coefficient of CMPs. We impose zero Neumann boundary conditions at both ends, assuming that CMPs neither enter nor leave the cell membrane. The initial condition is set as a normalized uniform distribution, $\rho_{\pm }(x,0)=1$.

### 2.3 Polarization of signaling cues by a GTPases model

To incorporate the complex interactions between Rho GTPase, specifically active and inactive forms of Rac1, Cdc42, and RhoA, as well as key phosphoinositides such as PIP (*P*_1_), PIP2 (*P*_2_), and PIP3 (*P*_3_), we take a model described in our previous publication on electrotaxis [50], which was based on several previously proposed models [51–56].

In summary, the active forms of Rac, Cdc42, and Rho are, respectively:


$$\begin{array}{c} \frac{\partial R}{\partial t}=-d_{R}R+D_{m}\partial_{x}^{2}R+(I_{R}+\alpha C)((1-f_{2})+f_{2}\frac{P_{3}}{P_{3b}})\frac{R_{i}}{R_{tot}}, \end{array}$$ (3)

$$\begin{array}{c} \frac{\partial C}{\partial t}=-d_{C}C+D_{m}\partial_{x}^{2}C+(\frac{I_{C}}{(1+(\frac{\rho^{A}}{a_{1}}{)}^{n}})((1-f_{1})+f_{1}\frac{P_{3}}{P_{3b}})\frac{C_{i}}{C_{tot}}\;\text{and} \end{array}$$ (4)

$$\begin{array}{c} \frac{\partial \rho^{A}}{\partial t}=(\frac{\left(I_{\rho^{A}}+\beta R\right)}{(1+(\frac{C}{a_{2}}{)}^{n}})\frac{\rho_{i}^{A}}{\rho_{tot}^{A}}-d_{\rho^{A}}\rho^{A}+D_{m}\partial_{x}^{2}\rho^{A}, \end{array}$$ (5)

respectively. The ${\text{C}}_{tot}$, ${\text{R}}_{tot}$, $\rho_{tot}^{A}$ are the total concentrations of the Cdc42, Rac and Rho. $I_{c},I_{R}$ and $I_{\rho^{A}}$ are activation rates, which we describe below. The values *a*_1_ and *a*_2_ are the Rho and Cdc42 concentrations that elicit a half-maximal drop of Cdc42 and Rho activation, respectively. The value α determines the rate of Cdc42-enhanced activation of Rac, and β determines the rate of Rac-enhanced Rho activation. *P*_3*b*_ is the baseline concentration of PIP3 found in a resting cell. $d_{R}$, $d_{C}$, and $d_{\rho^{A}}$ are the decay rates of activated Rac1, Cdc42 and Rho, respectively. $D_{m}$ is the diffusion constant of the active form. *f*_1_ and *f*_2_ are feedback from PIP_3_ to Cdc42 and Rac, respectively, which we assume differs from the original model [52], and they are bounded such that $0\leq f_{1},f_{2},\leq 1$. This allows us to analyze different feedback strengths from PIP3 to Rac1 and Cdc42.

$R_{i},C_{i},\rho_{i}^{A}$ are the concentrations of the respective inactive forms, which satisfy the equations:


$$\begin{array}{c} \frac{\partial R_{i}}{\partial t}-D_{mc}\partial_{x}^{2}R=-(I_{R}+\alpha C)((1-f_{2})+f_{2}\frac{P_{3}}{P_{3b}})\frac{R_{i}}{R_{tot}}+d_{R}R, \end{array}$$ (6)

$$\begin{array}{c} \frac{\partial C_{i}}{\partial t}-D_{mc}\partial_{x}^{2}C=-(\frac{I_{C}}{(1+(\frac{\rho^{A}}{a_{1}}{)}^{n}})((1-f_{1})+f_{1}\frac{P_{3}}{P_{3b}})\frac{C_{i}}{C_{tot}}+d_{C}C\;\text{and} \end{array}$$ (7)

$$\begin{array}{c} \frac{\partial \rho_{i}^{A}}{\partial t}=-\frac{\left(I_{\rho^{A}}+\beta R\right)}{(1+(\frac{C}{a_{2}}{)}^{n}}\frac{\rho_{i}^{A}}{\rho_{tot}^{A}}+d_{\rho^{A}}\rho^{A}+D_{mc}\partial_{x}^{2}\rho^{A}. \end{array}$$ (8)

${\text{D}}_{mc}$ is the diffusion coefficient of inactive Rho-proteins, which averages the respective diffusion rates of inactive GTPase forms by the time spent on the membrane versus the cytosol and diffuses much faster than the active forms (${\text{D}}_{m}$ ≪ ${\text{D}}_{mc}$).

Interactions between GTPases and PIPs are included in coupling terms between these sets of PDEs. The interaction between phosphoinositides, PIP (*P*_1_), PIP2 (*P*_2_), and PIP3 (*P*_3_), and between phosphoinositide kinases, are also modeled by a set of similar PDEs with reaction kinetics as


$$\begin{array}{c} \partial_{t}P_{1}-D_{p}\partial_{x}^{2}P_{1}=I_{P1}-\delta_{P1}P_{1}+k_{21}P_{2}-f_{PI5K}P_{1} \end{array}$$ (9)

$$\begin{array}{c} \partial_{t}P_{2}-D_{p}\partial_{x}^{2}P_{2}=-k_{21}P_{2}+f_{PI5K}P_{1}-f_{PI3K}P_{2}+f_{PTEN}P_{3} \end{array}$$ (10)

$$\begin{array}{c} \partial_{t}P_{3}-D_{p}\partial_{x}^{2}P_{3}=f_{PI3K}P_{2}-f_{PTEN}P_{3} \end{array}$$ (11)

where it is assumed that the conversion to PIP occurs at a constant rate, $I_{P1}$, and that all PIs diffuse in the membrane at a uniform rate $D_{p}$. $\delta_{P1}$ is the PIP_1_ decay rate and *k*_21_ is the PIP_2_ to PIP_1_ conversion rate. The model also describes the fact that Rac enhances the conversion of PIP to PIP2 (via PI5K), of PIP2 to PIP3 (via PI3K), and that Rho enhances the conversion of PIP3 to PIP2 via PTEN. The feedback is incorporated through the functions


$$\begin{array}{ccc} f_{PI5K} & = & \frac{k_{pi5k}}{2}(1+\frac{R}{R_{b}}), \end{array}$$ (12)

$$\begin{array}{ccc} f_{PI3K} & = & \frac{k_{pi3k}}{2}(1+\frac{R}{R_{b}}),\text{ and} \end{array}$$ (13)

$$\begin{array}{ccc} f_{PTEN} & = & \frac{k_{pten}}{2}(1+\frac{\rho^{A}}{\rho_{b}^{A}}), \end{array}$$ (14)

where ${\text{k}}_{PI3K}$ is the PIP_2_ to PIP_3_ baseline conversion rate, ${\text{k}}_{PI5K}$ is the PIP_1_ to PIP_2_ baseline conversion rate and ${\text{k}}_{PTEN}$ is the PIP_3_ to PIP_2_ baseline conversion rate. Whenever we modify ${\text{k}}_{pi3k}$, we also simultaneously adjust ${\text{k}}_{pten}$, as they are the forward and backward rates of the same reaction. This is done with $\rho_{-}$ and the same strength factor ${\text{S}}_{P}$. ${\text{R}}_{b}$, ${\text{C}}_{b}$ and $\rho_{b}^{A}$ are typical levels of active Rac, Cdc42 and Rho respectively.

We modified this model to incorporate stimuli arising from integrins and CMPs. To do so, we assume that the activation rates of the relevant signaling components depend on the magnitude of CMP accumulation or integrin activation. First, we assume that integrin engagement activates PI3K according to


$$\begin{array}{c} {\dot{k}}_{pi3k}=\lambda (S(\eta )-k_{pi3k})\;\text{with}\;S(\eta )=\frac{1}{1+e^{-10(\eta -0.6)}} \end{array}$$ (15)

an activation function that depends on the friction or, in other words, on the activity of cell adhesion following a sigmoidal function, where λ is a given relaxation rate that we fix to λ=0.5 to describe a slow biochemical adaptation. The slow adaptation accounts for chemical activation cascades, PI3K recruitment time to the membrane, and possible buffering events.

Regarding activation of Rho GTPases by polarized CMPs, we assume a linear relationship of the form


$$\begin{array}{c} I_{C}=\rho_{\pm }I_{C}^{*},\;I_{R}=\rho_{\pm }I_{R}^{*},\;k_{pi3k}=\rho_{\pm }k_{pi3k}^{*}, \end{array}$$ (16)

for Cdc42, Rac1, and PI3K activation, respectively. Here, the activation scales with the baseline conversion rates $I_{C}^{*}$, $I_{R}^{*}$, and $k_{pi3k}^{*}$ defined in the base model [52].

These modifications represent modeling assumptions that may require validation or refinement for specific cell types.

### 2.4 Mechanical model of the actomyosin network

To describe the mechanics of the actomyosin network, we adopt an active gel model [57,58], formulated as


$$\begin{array}{c} \partial_{x}\sigma =\eta v^{F}\;\text{in }\Omega . \end{array}$$ (17)

Here, inertial terms are neglected, and the internal stress σ is defined through a constitutive relation as


$$\begin{array}{c} \sigma =-2\mu \;\partial_{x}v^{F}+\zeta \;\rho^{A}\;\rho^{F}\rho^{M}, \end{array}$$

which accounts for both the viscous resistance of the actin cortex and the active contractility generated by the actomyosin system. In this expression, $\rho^{F}$ and $\rho^{M}$ represent the concentrations of F-actin and bound myosin motors, respectively, as detailed in the next section. The velocity $v^{F}$ corresponds to the actin network (or retrograde flow) measured in the laboratory frame. The parameter μ denotes the shear viscosity of the network, while ζ characterizes the active contractile stress generated by myosin motors. This contractility is further modulated by the local concentration of RhoA, thereby enhancing contractility in regions with elevated RhoA activity. Zero Neumann boundary conditions are imposed at both ends of the domain. The right-hand-side term in Eq. (17) represents the frictional interaction between the moving cortex and its surrounding environment. We assume this friction to be linearly proportional to the cortex velocity, with coefficient η [29,58], as defined above.

### 2.5 Kinetics of the cell fronts

The polymerization velocity of actin filaments at the cell front is biochemically regulated by the activity of Rac1 and Cdc42, which modulate Arp2/3 complex activation and filament nucleation [22]. In the absence of opposing forces, the polymerization velocity is defined as


$$\begin{array}{c} v_{0}^{p}=k_{on}\;\delta \;R, \end{array}$$

where $k_{on}$ represents the actin assembly rate at the leading edge, and δ denotes the size of a single actin monomer. Because $v_{0}^{p}$ depends on the concentration of active Rac1, *R*, actin protrusion is enhanced at the cell front, where Rac1 is abundant, and inhibited at the rear.

Polymerization is physically opposed by the membrane tension, τ(L(t)). We assume that membrane tension follows a linear relationship given by


$$\begin{array}{c} \tau =-k\left(L(t)-L_{0}\right), \end{array}$$

where *k* denotes the membrane stiffness and *L*_0_ is the initial cell length, and the cell length is defined as $L(t)=|l_{l}(t)-l_{r}(t)|$.

To describe the dependence of polymerization on membrane tension, we adopt a well-established model introduced by [59], in which the polymerization velocity decreases with increasing tension according to


$$\begin{array}{c} v^{p}=v_{0}^{p}{\left[1-\frac{\tau (L(t))}{\tau_{stall}}\right]}^{\gamma }, \end{array}$$ (18)

where $\tau_{stall}$ represents the membrane tension required to stall polymerization, and γ determines how sharply the velocity decays with increasing tension.

Given both the retrograde actin flow velocity, *v*, and the actin polymerization velocity, $v^{p}$, the motion of the cell boundaries is obtained as a combination of inward flow and outward protrusion. Specifically, the velocities of the front and rear edges are defined as


$$\begin{array}{c} {\dot{l}}_{f}(t)=v_{f}+v_{f}^{p},\;\text{and}\;{\dot{l}}_{b}(t)=v_{b}+v_{b}^{p}, \end{array}$$

respectively. The net migration velocity of the cell is then given by


$$\begin{array}{c} v=\frac{{\dot{l}}_{f}(t)+{\dot{l}}_{b}(t)}{2}. \end{array}$$ (19)

### 2.6 Transport of the actomyosin network

Finally, to model the density dynamics of the actomyosin network, we employ a coupled convection–diffusion framework to describe the spatiotemporal evolution of filamentous (F-actin), $\rho^{F}(x,t)$, and globular (G-actin), $\rho^{G}(x,t)$, actin species within the cell (see [58] and references therein):


$$\begin{array}{ccc} \partial_{t}\rho^{F}+\partial_{x}\left(w\rho^{F}-D^{F}\partial_{x}\rho^{F}\right) & = & k_{p}\rho^{G}-k_{d}\rho^{F}, \end{array}$$ (20)

$$\begin{array}{ccc} \partial_{t}\rho^{G}-\partial_{x}\left(D^{G}\partial_{x}\rho^{G}\right) & = & k_{d}\rho^{F}-k_{p}\rho^{G}. \end{array}$$ (21)

Here, $D^{F}$ and $D^{G}$ denote the diffusion coefficients of F-actin and G-actin, respectively, while $k_{p}$ and $k_{d}$ are the polymerization and depolymerization rates. Because G-actin diffuses much more rapidly than it is advected by flow, convection is neglected in its equation. The retrograde flow relative to the cell frame is defined as $w=v^{F}-v$. Zero-flux boundary conditions are imposed at both cell edges, assuming that neither form of actin crosses the membrane boundary.

In addition, we consider a two-species model for myosin motors: a bound form, $\rho^{M}$, attached to the F-actin network, and an unbound form, $\rho^{m}$. Their redistribution is similarly governed by convection–diffusion dynamics:


$$\begin{array}{c} \partial_{t}\rho^{M}+\partial_{x}\left(w\rho^{M}-D^{M}\partial_{x}\rho^{M}\right)=k_{b}^{M}\rho^{m}-k_{u}^{m}\rho^{M}, \end{array}$$ (22)

$$\begin{array}{c} \partial_{t}\rho^{m}-D^{m}\partial_{x}^{2}\rho^{m}=k_{u}^{m}\rho^{M}-k_{b}^{M}\rho^{m}. \end{array}$$ (23)

Here, $D^{M}$ and $D^{m}$ denote the diffusion coefficients of bound and unbound myosin, respectively, while $k_{b}^{M}$ and $k_{u}^{m}$ represent the binding and unbinding rates of myosin to F-actin. As with G-actin, the diffusivity of unbound myosin is assumed to dominate, allowing convection to be neglected in Eq. (23). Zero-flux boundary conditions are again applied at both ends of the domain to ensure that neither form of myosin enters nor leaves the cell.

### 2.7 Numerical solution of the problem and model parameters

We numerically solve all coupled PDE systems using a staggered approach. Spatial discretization is performed using the Finite Element Method (FEM), while temporal discretization of the parabolic equations is carried out using the implicit, second-order Crank–Nicolson scheme [60]. Nonlinear equations are solved iteratively via the Newton–Raphson method. Unless otherwise specified in the main text, all simulation parameters are adopted from previously published studies [29,50,58].

## 3 Results

**Fig 1:**
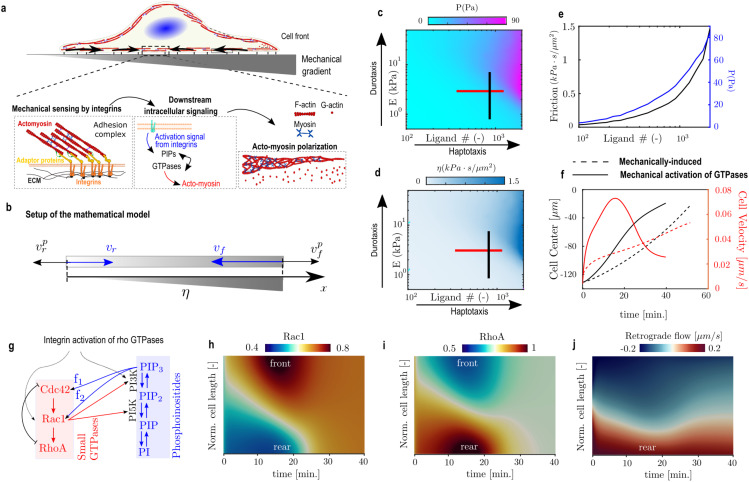
Mechanotaxis in single-cell migration. **(a)** Schematic of the main mechanisms involved in mechanotaxis, including integrin activation, downstream signaling, and actomyosin polarization. **(b)** Key variables in the physical model: retrograde flow velocity *v* (blue), indicated at the front and rear of the cell (subscripts *f* and *r*, respectively), and actin polymerization velocity (black). **(c-d)** Traction forces (c) and corresponding effective friction (d) in response to gradients in ECM stiffness and anchoring point density. Red and black lines represent traction and friction gradients of similar magnitude for haptotaxis and durotaxis, respectively. **(e)** A representative traction and friction profile used in the simulations. **(f)** Time evolution of cell center position (black line) and migration velocity (red line) in two conditions: (1) when mechanotaxis is driven solely by a friction gradient (dashed lines), as described in [29], and (2) when integrin activation also triggers downstream activation of PI3K and subsequently Rho GTPases (solid lines). Incorporating biochemical signaling results in faster cell migration, due to increased actin polymerization at the front and enhanced retrograde flow at the rear of the cell. **(g)** Schematic of the Rho GTPase signaling network included in the mathematical model. Arrows indicate positive feedback (activation) from one component to another, and tails indicate negative feedback (inactivation) of one component by another. *f*_1_ and *f*_2_ are the magnitudes of feedback for activating Cdc42 and Rac1 by PIP3. **(h-j)** Kymographs of variables along the normalized cell length (Y-axis) over time (X-axis) when mechanical activation of GTPases is included in the mechanotactic model: Rac1 **(h)**, RhoA **(i)**, and retrograde flow velocity **(j)**. In all kymographs, the positions of the cell front and rear are indicated.

To investigate how simultaneous mechanical and electrical stimulation influences cell migration, we largely follow the approach presented in our previous publications [29,50,58], which uses a minimal active gel model to characterize the dynamics of the actomyosin network by solving the transport equations for actomyosin density in conjunction with the momentum balance equation for the retrograde flow. To model the cascade of events leading to mechanotaxis and electrotaxis, we consider the primary cellular sensors involved, integrins and CMPs, respectively, and their activation of intracellular signaling networks [29,50]. In short, we consider a one-dimensional computational model, represented as a continuous segment with moving boundaries $x(t)\in [l_{l}(t),l_{r}(t)]$, where $l_{l}(t)$ and $l_{r}(t)$ denote the positions of the left and right edges of the cell, respectively. The model computes the retrograde velocity *v* and the polymerization velocity against the cell membrane $v^{p}$, from which we calculate the motion of the cell boundaries as a combination of inward retrograde flow and outward protrusion such that the velocities of the front and rear edges are given by ${\dot{l}}_{f}(t)=v_{f}+v_{f}^{p}$ and ${\dot{l}}_{b}(t)=v_{b}+v_{b}^{p}$, respectively. Then, the migration velocity of the cell is given as $v=({\dot{l}}_{f}(t)+{\dot{l}}_{b}(t))/2$ (see Fig 1A).

If no symmetry-breaking is induced, the cell will show symmetric flows and actomyosin densities, and, consequently, cell spreading [61–63]. This non-migrating, static cell mode has been reproduced using such active gel models [58] and also used to describe the transmission of forces between different cellular networks and adhesion structures [64].

During mechanotaxis, gradients in mechanical cues of the ECM induce asymmetric expression of cell adhesions, highly dynamic structures primarily mediated by transmembrane receptors such as integrins, which play a crucial role in guiding directed migration. The clutch model [42] has been instrumental in explaining and predicting cell adhesion behavior across various ECM conditions, including elastic [45,65], viscoelastic [66,67], and ligand density [68] (see [43] for a detailed overview). By coupling an active gel model of cell migration with a stochastic clutch framework, it is possible to describe how the active forces of the cell are transmitted asymmetrically outside the cell, inducing cell movement. This mathematical approach has been used, for example, to describe both positive and negative durotaxis across different cell types [29]. To develop our current model, we begin with a simplified version of this previous durotactic model [29].

First, we compute the traction forces using the clutch model, along with the effective friction (see Section 2.1 for details) resulting from haptotactic and durotactic cues (Fig 1C–1D). We then extract the traction and friction profiles corresponding to specific haptotactic and durotactic gradients (represented by the red and black lines in Fig 1C–1D), and impose these gradients over a substrate of 250 μm in length. Therefore, rather than dynamically solving the clutch model over time, we apply the precomputed effective friction along each position of the cell–substrate interface. To initialize our simulations, we assumed a cell with constant normalized acto-myosin density, zero retrograde flow, and placed the cell center at -125 μm

As previously demonstrated, cells migrate along friction gradients (Fig 1F) [29], rather than stiffness gradients alone—challenging earlier assumptions and offering a theoretical rationale for previously observed phenomena described as positive and negative durotaxis. However, this durotactic model predicts a lower migration velocity compared to experimental observations [69,70]. One possible explanation is that the model accounts solely for the mechanical interactions mediated by cell adhesions, while neglecting the fact that, as discussed above, integrin activation also initiates Rho GTPase signaling, which could enhance the cell’s migration speed.

Integrin activation involves a conformational transition from a low-affinity (inactive) state to a high-affinity (active) state, regulated by intracellular adaptor proteins such as talin and kindlin. This process is controlled by bidirectional signaling: intracellular cues regulate integrin activation (inside-out signaling), while integrin–ECM interactions transmit signals back into the cell (outside-in signaling), influencing cytoskeletal organization and downstream signaling pathways. Notably, integrin engagement activates PI3K [71–74]. In fibroblasts, adhesion to fibronectin has also been shown to promote transient Rac activation and sustained Rho activation [75–79]. Other studies have reported that integrin-mediated adhesion activates both Cdc42 and Rac1 [77,80], while some suggest that Cdc42 is activated first and subsequently triggers Rac activation [78].

To incorporate these activation mechanisms, we coupled our mechanotactic model with a well-established Rho GTPase signaling model [51–53] This model includes both active and inactive forms of Rac1, Cdc42, and RhoA, as well as key phosphoinositides: PIP (*P*_1_), PIP2 (*P*_2_), and PIP3 (*P*_3_) (see Fig 1G). To account for the activation of Rho GTPases by integrins, we follow a previous model [50] and consider that the conversion of PIP2 to PIP3 via PI3K depends on integrin activation, which we assume to be proportional to friction. Following experimental insights [71–74], we assumed that higher frictional forces—indicative of greater integrin engagement and traction—lead to increased PI3K activation rates.

This signaling pathway influences the actomyosin network in two ways. First, the force generated by myosin motors, ξ, is made linearly dependent on the local concentration of active RhoA, such that regions with high RhoA exhibit stronger contractile forces, while areas with low RhoA show weaker myosin activity [13–15]. Second, the tension-free actin polymerization velocity at the leading edges of the cell, $v^{p}$, is assumed to vary linearly with Rac1 density, meaning that actin polymerization is enhanced in regions where Rac1 accumulates [16–19].

We then repeated the simulations described previously, now incorporating these additional biochemical interactions. The model reveals dynamic changes in Rac1 and RhoA levels, with both polarizing toward the friction gradient (see Fig 1H–1I). As a consequence, the retrograde flow becomes further polarized (Fig 1J). We have assumed a sigmoidal-like response of PI3K activation due to friction, which saturates at high friction values. Hence, once the cell reaches a region of high friction, the activation of PI3K by integrins becomes uniform from front to rear, as well as Rac1 and RhoA (Fig 1H–1I). Altogether, the enhanced model predicts a higher migration speed compared to a cell influenced by a friction gradient alone (Fig 1F).

**Fig 2:**
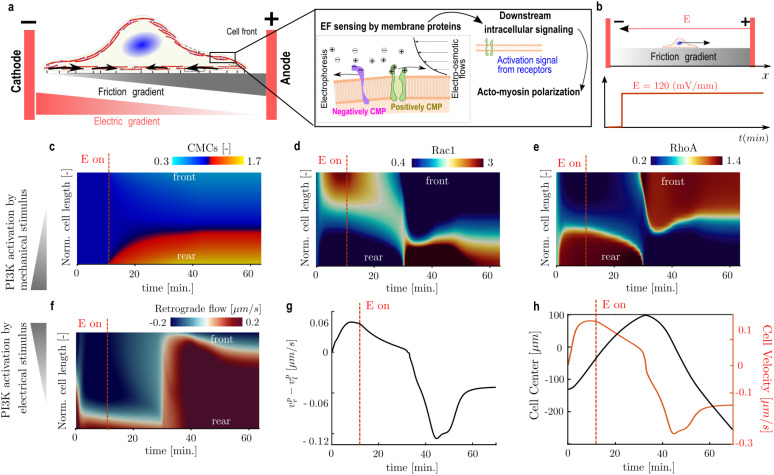
Interplay between mechanotaxis and electrotaxis in single cell migration. **(a)** Opposing cues and sketch of the main mechanisms involved in electrotaxis: activation of CMPs, downstream signaling, and actomyosin polarization. **(b)** Sketch of the opposing activation of the electric field as the cell migrates along a friction gradient. **(c-h)** Polarization of CMPs induces a downstream activation of PI3K and, consequently, of the Rho GTPases. **(c-f)** Kymograph of model variables along the normalized cell length (Y axis) over time (X axis). The kymographs show CMPs **(c)**, Rac1 **(d)**, RhoA **(e)**, and the retrograde flow **(f)**. **(g)** Difference in the polymerization velocity of actin against the cell membrane. **(h)** The position of the cell center (black lines) and migration velocity of the cell (red) is shown.

Next, we extend this successful mechanotactic model to examine the interplay between electrical and mechanical cues and how they compete to direct cell migration (Fig 2A). Specifically, our goal is to determine which cue—mechanical or electrical—is more effective in guiding cell migration. During electrotaxis, the electromigration of CMPs, from both electrophoretic and electroosmotic effects, leads to a polarized and enhanced activation of GTPases [36,39,81–85]. Therefore, both mechanotaxis and electrotaxis rely on the polarization of intracellular signaling pathways that regulate the cell’s motility machinery (Fig 2A–2B). To address this interplay, we integrated the GTPase-based mechanotactic model described above, which is driven by mechanical stimulation, with our previously developed electrotaxis model (see section 2 and [50] for details), also employing the same GTPase signaling framework. Among the various possible downstream activations by CMPs, we first assume activation of PI3K by the electrically polarized CMPs [83,84].

We apply the same stiffness gradient, position the cell center at −125μm, and allow the cell to migrate, as before. Thereafter, when the leading edge of the cell reaches x=−20μm, we activate an EF of 120mV/μm, with the cathode positioned at the rear of the migrating cell (Fig 2B). Therefore, a stiffness gradient and an EF in the opposite direction are being applied. During this period, signals from the frictional gradient and CMP polarization toward the anode compete, leading to opposing stimuli for the activation of the signaling layer (see Fig 1G) and, eventually, for the polarization of the cell.

Once the EF is applied, CMPs redistribute rapidly, on the order of seconds (Fig 2C). There is an additional delay between EF activation and the subsequent change in migration direction, due to the turnover dynamics of PIPs and Rho GTPases (Fig 2D–2E). Specifically, RhoA-driven actomyosin flow (Fig 2F) and Rac1-driven protrusive polymerization are modulated (Fig 2G). As a result, we observe a change in migratory direction (Fig 2H), indicating that electrotaxis can revert the migration along the stiffness gradient. For this specific set of model parameters, or cell-type, the migration velocity stabilizes after 70 min, indicating that the opposing activations of PI3K balance each other (Fig 2H).

**Fig 3:**
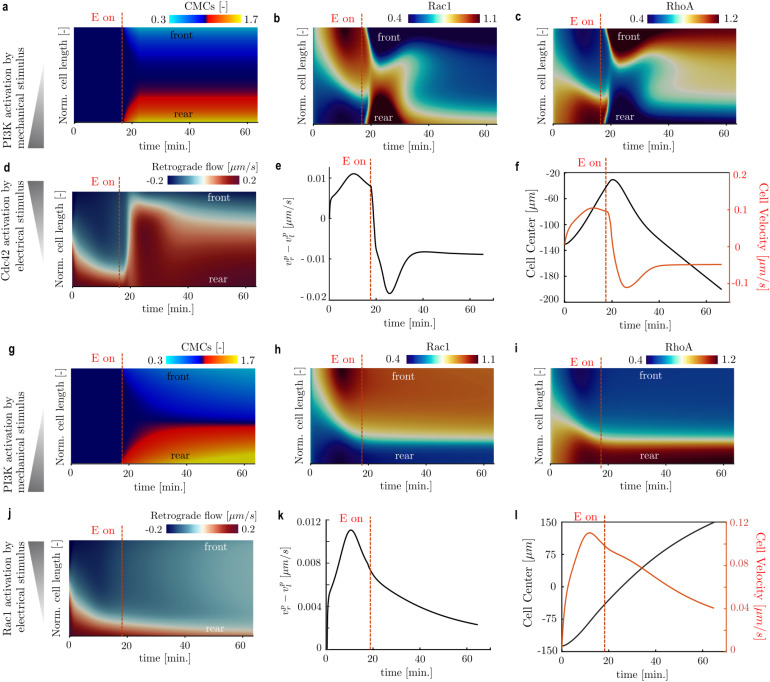
Polarization of CMPs induces a downstream activation of Rac1 and, consequently, of the Rho GTPases. **(a-d)** Kymograph of model variables along the normalized cell length (Y axis) over time (X axis). The kymographs show CMPs **(a)**, Rac1 **(b)**, RhoA **(c)**, and the retrograde flow **(d)**. **(e)** Difference in the polymerization velocity of actin against the cell membrane. **(f)** The position of the cell center (black lines) and migration velocity of the cell (red) is shown. In all kymographs, we indicated the position of the front and rear of the cell. **(g-l)** Polarization of CMPs induces a downstream activation of Rac1 and, consequently, of the Rho GTPases. **(g-j)** Kymograph of model variables along the normalized cell length (Y axis) over time (X axis). The kymographs show CMPs **(g)**, Rac1 **(h)**, RhoA **(i)**, and the retrograde flow **(j)**. **(k)** Difference in the polymerization velocity of actin against the cell membrane. **(l)** The position of the cell center (black lines) and migration velocity of the cell (red) are shown.

There are also data indicating that activation by CMPs may directly stimulate the activation of Rac1 and Cdc42 [86–89]. To analyze these possibilities, we first recompute the previous simulation with the direct activation of Cdc42 by CMPs instead of PI3K (Fig 3A–3F). Results are similar to the PI3K-based activation. However, we observe a re-polarization of Rac1 and RhoA (Fig 3B–3C), instead of the uniform distribution obtained for the PI3K-based activation, because the effect of the electric stimuli is larger than the PI3K-based activation. This polarization also creates a re-polarization of the retrograde flow (Fig 3D), which is, in part, responsible for the reversal in migration direction (Fig 3E). The cell reaches a steady migration velocity toward the cathode (Fig 3F).

We also recompute the simulation with the activation of Rac1 by CMPs (Fig 3G–3L). We do not observe migration reversal but a reduction of migration speed (Fig 3L) during the same period of time as in the previous simulation. After the initial polarization of Rac1 and RhoA (Fig 3H–3I), and consequently of the retrograde flow (Fig 3J), we show a steady state. These results indicate that activation of Rac1 by the EF, as opposed to the PI3K activated by a mechanical stimulus, is not enough to change the migration direction, and it just acts to reduce the migration capacity. Therefore, Rac1 seems to be a weaker effector for directed cell migration.

**Fig 4:**
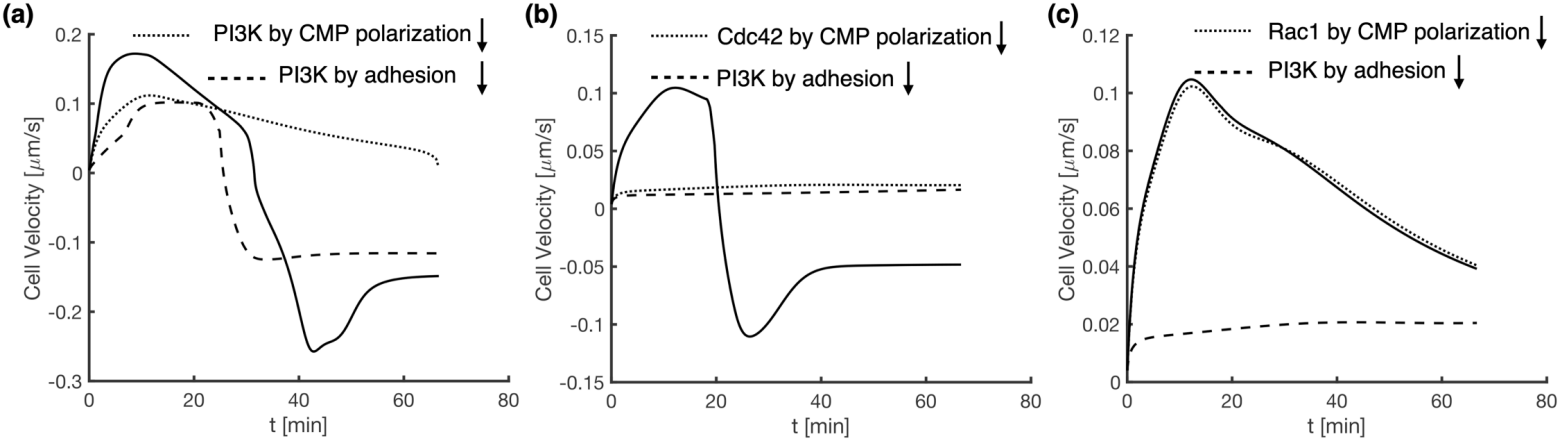
Parametric analysis of the feedback strengths associated with integrin activation and CMP polarization. **(a)** Relative to the control case shown in Fig 2 (solid line), the feedback from integrin activation to PI3K activation is reduced to 10% of its original value (dashed line), and the feedback from CMP stimulation to PI3K activation is reduced to 10% of its original value (dotted line). **(b)** Relative to the control case shown in Fig 3 (solid line), the feedback from integrin activation to PI3K activation is reduced to 10% of its original value (dashed line), and the feedback from CMP polarization to Cdc42 activation is reduced to 10% of its original value (dotted line). **(c)** Relative to the control case shown in Fig 3 (solid line), the feedback from integrin activation to PI3K activation is reduced to 10% of its original value (dashed line), and the feedback from CMP polarization to Rac1 activation is reduced to 10% of its original value (dotted line).

Because our model depends on the coupling between cell adhesion and PI3K activation, as well as on the polarization of CMPs and the activation of downstream signaling pathways, we performed a parametric analysis of these feedback strengths (Fig 4). A detailed parametric analysis of the signaling model and the active gel model independently can be found elsewhere [50,58]. Here, we focus on the three cases presented in Figs 2–3.

First, we examined the effect of reducing the feedback from integrin activation and CMP stimulation on PI3K activation (Fig 4A). The results show that reducing the feedback from integrin activation preserves the shift in migration direction observed in the baseline case but decreases the magnitude of the migration velocity. In contrast, reducing the feedback from CMPs to PI3K activation does not induce a shift in migration direction, and cells continue to migrate toward the positive friction gradient.

Next, we investigated the effect of reducing the feedback from integrin activation to PI3K activation and from CMP polarization to Cdc42 activation (Fig 4B). The results indicate that reductions in both feedback strengths produce similar effects on cell migration velocity, decreasing it to approximately 0.02 μm/s. Furthermore, no reversal of migration direction is observed, suggesting that direct activation of Cdc42 and PI3K plays a dominant role in regulating cell migration and is insufficient to overcome the underlying mechanotactic response.

Finally, we analyzed the effects of reducing the feedback from integrin activation and CMP stimulation on Rac1 activation (Fig 4C). The results show that decreasing Rac1 activation has only a minor effect on migration velocity, whereas reducing PI3K activation downstream of integrin signaling again lowers the migration velocity to approximately 0.02 μm/s, consistent with the previous results. Overall, these findings emphasize that variations in model parameters, or, equivalently, differences in the signaling behavior of distinct cell types, can lead to substantial changes in migration velocity and, in some cases, even alter the direction of migration.

## 4 Discussion

Overall, our results show how the interplay between electrical and mechanical stimuli in guiding cell migration arises from the activation of GTPase signaling through integrins or membrane proteins, and that the relative strength of these activation pathways ultimately determines which cue prevails. For the specific model parameters used in this work, and also for variations in our parametric analysis, our model results suggest that PI3K and Cdc42 are the main regulators in orchestrating the signaling state of GTPases in the cell and, therefore, symmetry breaking, polarity, and directional migration of the cell.

At this point, it became evident that determining whether mechanical or electrical stimulation dominates over the other is not a universal question but one that likely depends on the specific cell type. Although our model integrates the main mechanisms underlying mechanotaxis and electrotaxis, it involves a large number of parameters that strongly determine the effect of each Rho GTPase over the others in a cell-specific manner. For example, our model results suggest that leading-edge lamellipodial protrusions are inherently weaker than RhoA-mediated trailing-edge contractility when dictating whole-cell directional steering. However, changes in the model parameters and the signalling model construction would affect these conclusions. One possible line of future work is the parametric analysis of all model parameters, although those have been done in the past for the three mechanistic layers in the taxis procedure. We believe that the understanding of each specific cell line cannot be fully resolved without complementary experimental validation, and that should be the focus of future research. The key outcome of our modeling effort is the identification, formulation, and validation of specific experimental strategies needed to address this issue in a cell-type-dependent manner.

As we discussed, central to both types of stimuli is the GTPase signaling network, which serves as a convergence point for upstream cues and downstream effects [11,12]. In this work, we have adopted a well-established signaling framework that captures the interactions between GTPases and their regulators [51–53]. However, variations in feedback strengths, activation-deactivation kinetics, and receptor coupling are expected across different cell types. Fortunately, these aspects are readily available through mathematical modeling, and our model can provide predictions at minimal cost that can be later explored experimentally.

The first step in dissecting mechanotactic and electrotactic behavior should thus focus on characterizing the signaling profile of the cell under study, specifically on integrin forms and all membrane components responsible for the GTPases signaling [11,90,91]. Indeed, different integrin subunits have been demonstrated to have significant regulatory roles in basal motility of cells, durotaxis and haptotaxis, and electrotaxis, albeit separately [92–94]. Selective expression of integrin subunits and membrane components could determine the migration direction of cells in an electric field, mediated by PI3 kinase signaling [94]. Thus, to test directly the role of integrin subunits and membrane proteins that regulate PI3K and GTPases to mechanical and electrical cues, one can manipulate the expression of these key membrane proteins with tunable CRISPR-interference (CRISPRi) or photoactivatable cas-13 [95]. This would allow for a precise spatio-temporal perturbation while assessing semi-quantitatively the contribution of each particular integrin subunit and membrane protein. These datasets would help in the experimental design of targeted perturbations and identify the upstream components most relevant for a given context. In the context of electrotaxis, preliminary screening of CMPs [49,50] and integrins [94] has been attempted, but must be more closely investigated.

One global approach would require the tracking of membrane microdomains (rafts), which are composed of specific lipid environments and CMPs such as receptors and ion channels. Note that in a multicellular system, the connection between cells can create regions of local barriers, which could limit the movement of CMPs. Even if we do not know what proteins comprise these rafts at first, the indication that the lipid rafts have a compound nominal charge, because they have many identical proteins together, means that the raft can easily be “more positive” or “negative” and thus be responsive to the electric field [96]. Moreover, rafts have specific lipids like cholesterol that are uncharged, which can make the membrane more fluid-like because there’s less electrostatic interaction with lipid tails and thus less compaction. Markers for lipid rafts like Filipin III and CTxB can be used to understand whether polarization of CMPs is dependent on protein-lipid complexes or whether individual components are sensing the EF and responding through electrophoresis and electro-osmotic flows. Other candidate molecules, such as EGFR or VEGFR, could be labeled for single-molecule microscopy, to be tracked during EF activation and determine which CMPs respond and repolarize to an EF. Another approach would be to knock down electrosensors such as VSP1, Galvanin (TMEM154), and PTEN and study the effect on electrotaxis direction [38,97–99].

To further test our models regarding PI3K, an optogenetically controlled PI3K can be used to control its activity and that way, create an opposing signal to either mechanotaxis or electrotaxis [100]. Another way to test how determinant PI3K is for polarity and onset of migration is to use cell lines depleted for PI3K or use inhibitors, and check whether directionality is maintained with the biasing cues.

One outstanding question is whether PIPs are passively displaced by membrane flows upon EF activation. Therefore, tracking PIP2 or PIP3 fluorescent markers during EF activation could reveal whether PIPs are physically drifted or synthesized de novo at emerging polarity sites, and potentially whether the EF directly mobilizes the enzymatic machinery responsible for PIP phosphorylation and dephosphorylation. Since GTPases have a complex affinity to bind PIP kinases, PIP2, or even require PIP2 to activate their effectors, GTPases and PIP dynamics should be tracked in parallel to understand these relationships at the onset of cell repolarization. Many of these experiments or experimental strategies have already been applied in chemotaxis, hence a great inspiration and set of toolboxes can be easily adapted from there [101]. Besides these ideas, if one truly aims to understand how multimodal cues work in additive, synergistic, or confronting manners, a key direction is to characterize the environment in which cells migrate. This will deeply benefit theory and experimental design as it will report on the native scenarios in which cells move. For example, one can design experiments in which orthogonal, parallel, or antiparallel mechanical and electrical gradients, similar to those presented above, are applied while monitoring the parameters described above.

In the case of mechanical inputs, traction force microscopy paired with controlled substrate stiffness or micropatterned substrates [93] can be used to quantify how specific integrins modulate GTPase activation dynamics. Furthermore, to determine what integrins and CMPs, and at what strength and timing, activate which layers of the GTPase cascade, time-resolved imaging of integrin and CMPs clustering and associated GEF/GAP activity would be essential. This can be approached by live-cell FRET biosensing to monitor activities of Rac1, Cdc42 and Rho in real time during durotaxis/haptotaxis and upon the onset of an EF [102,103], which can be combined with genetic perturbation (e.g., CRISPR-Cas9-mediated knockout or overexpression of specific integrins/CMPs). The onset of an EF can be controlled in direction and magnitude of the applied EF in an electrotaxis chamber, which allows direct test and validation of the modelling results as shown in Figs 1–3. These experiments will directly compare the model dynamics of GTPase dynamics and the changes upon an EF being switched on.

Finally, how each Rho GTPase quantitatively influences actomyosin contractility has been much further investigated. For example, a common implementation consists of the use of myosin II fluorescent reporters (e.g., MRLC-GFP), in conjunction with pharmacological modulation of individual GTPases (using inhibitors or constitutively active mutants) and time-lapse confocal microscopy [104,105]. Quantitative image analysis would then reveal how each GTPase contributes to cytoskeletal contractility in a stimulus-dependent manner. More importantly, and going back to the idea of synergy, addition or competition, the contribution of piezo electric membrane channels, such as TRPs or Piezos, which can rapidly deliver calcium influxes due to changes in membrane voltage and stretch, will also contribute to dissecting the molecular mechanism by which cells sense and translate electromechanical stimuli into a cellular pattern of actomyosin contractility.

We must also mention that the present framework is restricted to a one-dimensional description of migration, which necessarily limits its ability to capture the full extent of stochasticity observed in experimental systems [4,106]. A 2D formulation including sources of stochasticity would restore these degrees of freedom, allowing stochasticity to emerge through variability in directionality and spatial exploration, and thereby provide a more faithful representation of single-cell migration dynamics. Understanding the combined effect of external cues should also be investigated in the context of multicellular systems [40,70,107].

Together, these experimental strategies would provide the necessary resolution to determine, for any given cell, which stimulus exerts dominant control over directional migration. Integrating these cues could lead to the development of efficient and controllable strategies for guiding cell migration, with significant implications for limiting cancer invasion, promoting wound healing, and engineering structured tissues on demand.
